# Immature functional development of lumbar locomotor networks in adult *Irf8^−/−^* mice

**DOI:** 10.3389/fnins.2023.1234215

**Published:** 2024-01-04

**Authors:** Itaru Yazawa, Yuko Yoshida, Ryusuke Yoshimi, Keiko Ozato

**Affiliations:** ^1^Department of Food and Nutrition, Kyushu Nutrition Welfare University, Kitakyushu, Japan; ^2^Laboratory of Neural Control, National Institute of Neurological Disorders and Stroke (NINDS), National Institutes of Health (NIH), Bethesda, MD, United States; ^3^Division of Developmental Biology, National Institute of Child Health and Human Development (NICHD), NIH, Bethesda, MD, United States; ^4^Department of Stem Cell and Immune Regulation, Graduate School of Medicine, Yokohama City University, Yokohama, Japan

**Keywords:** adult interferon regulatory factor 8 (IRF8)-deficient mice, decerebrate and arterially perfused *in situ* preparations, immature adult lumbar CPG, discharge episodes, rhythmic discharge

## Abstract

To date, research on the role of the brainstem and spinal cord in motor behavior has relied on *in vitro* preparations of the neonatal rodent spinal cord, with or without the brainstem; their spatial and temporal scope are subject to technical limitations imposed by low oxygen tension in deep tissues. Therefore, we created an arterially perfused *in situ* preparation that allowed us to investigate functional interactions in the CNS from the neonatal to adult period. Decerebrated rodents were kept alive via total artificial cardiopulmonary bypass for extracorporeal circulation; the plasma oxygen and ion components needed for survival were supplied through the blood vessels. Interferon regulatory factor 8 (IRF8) is a transcription factor that promotes myeloid cell development and stimulates innate immune responses. In the brain, IRF8 is expressed only in microglia and directs the expression of many genes that serve microglial functions. Recent evidence indicates that IRF8 affects behavior and modulates Alzheimer’s disease progression in a mouse model. However, whether this immune deficiency arising from the absence of IRF8 influences the development of the neuronal network in the spinal cord is unknown. We applied the above methodology to mice of all ages and electrophysiologically explored whether the absence of IRF8 influences the development of lumbar central pattern generator (CPG) networks. In mice of all ages, bilateral neuronal discharges by the normal CPG networks activated by the modulated sympathetic tone via descending pathways at high flow rates became organized into discharge episodes punctuated by periods of quiescence. Similar discharge episodes were generated by the adult CPG networks (≥P14 days) activated by drug application. However, discharge episodes elicited by activating the neonatal-juvenile CPG networks (<P14 days) occurred alternately on the left and right sides. Interestingly, discharge episodes elicited by the CPG networks in adult IRF8 knockout mice (P11–12 weeks) consisted of those elicited by the CPG networks of both periods. Thus, it was suggested that growing up with immunodeficiency due to loss of IRF8 might interfere with the normal development of functions exerted by the lumbar CPG network because IRF8 plays a role in the normal development of the lumbar CPG network.

## Introduction

To date, most research on the role of the brainstem and spinal cord in motor behavior has relied on *in vitro* preparations of the neonatal rodent spinal cord, with or without the brainstem; their spatial and temporal scope are subject to technical limitations imposed by low oxygen tension in deep tissues (St John 1985; Brockhaus et al., 1993; Wang et al., 1996; Wilson et al., 2003; Fong et al., 2008). To overcome this difficulty, we modified the arterially perfused *in situ* preparation, originally developed by Pickering and Paton (2006), which allows us to investigate functional interactions in the central nervous system (CNS), especially between the brainstem and the lower spinal cord, from the neonatal to adult period. This preparation can be used to explore unknown autonomous functions and provide clues to their mechanisms, as well as to track functional changes in the CNS around critical periods. In this methodology, decerebrated mice were kept alive via total artificial cardiopulmonary bypass for extracorporeal circulation; the plasma oxygen and ion components needed for survival were supplied by blood vessels (Yazawa, 2014).

Interferon regulatory factor 8 (IRF8) is a transcription factor that promotes myeloid cell development and stimulates innate immune responses (Tamura et al., 2000; McLellan et al., 2002; Tamura and Ozato, 2002; Yamanaka et al., 2008). In the brain, IRF8 is expressed only in microglia and directs the expression of many genes that serve microglial functions (Kierdorf et al., 2013). Microglia play a role in regulating the number of neural stem cells from the embryo to the postnatal stage by inducing the apoptosis of unnecessary neural stem cells and neurons and engulfing them (Cunningham et al., 2013; Brown and Neher, 2014). In the process of neural circuit formation after the postnatal stage, microglia contribute to the functional maturation of neural circuit formation by retaining only the necessary synapses among the excess synapses and eliminating the unnecessary synapses (Schafer et al., 2012; Brown and Neher, 2014). IRF8-deficient mice (*Irf8^−/−^* mice), in which macrophages and microglia are defective in functions, including cytokine production, are known as an animal model for human chronic myeloid leukemia, in which granulocytes (neutrophils) are systemically increased (Holtschke et al., 1996); these mice are recognized as a vital tool for studying the immunological events related to the disease. Masuda et al. showed that microglia expressing IRF8 in the lumbar cord dorsal horn increase after peripheral nerve injury and that IRF8 is needed for cutaneous tactile allodynia, and the perception of pain, revealing that IRF8 in microglia affects neuronal morphology and function (Masuda et al., 2012). Furthermore, it has been shown that IRF8 is expressed in microglia from the embryonic stage and throughout adulthood at similar levels and is thought to direct the development and maintenance of neuronal networks (Kierdorf et al., 2013; Saeki et al., 2023).

However, whether the loss of IRF8-related microglia resulting from the absence of IRF8 influences the development of the neuronal network in the lumbar spinal cord is unknown.

In this study, the above methodology was applied to mice of all ages, and the interplay of the discharge episodes from the left and right peripheral motor nerves resulting from the activation of the lumbar central pattern generator (CPG) networks was examined using electrophysiological techniques to explore whether the absence of IRF8 influences the development of lumbar CPG networks.

## Materials and methods

### Subjects

Ten female wild-type (WT) mice and 10 female *Irf8^−/−^* mice on a C57BL/6 background (Jackson Laboratories), aged 11–12 weeks and weighing 15.5–21.4 g, were used in this study, along with 20 male Swiss Webster mice (Taconic Laboratory) aged 5–51 days and weighing 4.1–45.2 g. The experimental protocols were approved by the National Institute of Neurological Disorders and Stroke (NINDS) and the National Institute of Child Health and Human Development (NICHD)/National Institutes of Health (NIH) Animal Care and Use Committee.

### Decerebrate and arterially perfused *in situ* mouse preparation

Experiments were performed on 5 female WT and 5 female *Irf8^−/−^* mice on the C57BL/6 background (Jackson Laboratories) aged 11–12 weeks and 10 male Swiss Webster mice (Taconic Laboratory) aged 5–21 days. Mice were sedated by inhalation anesthesia with 5.0% halothane and were intraperitoneally injected with an anesthetic combination of ketamine and xylazine (0.5–1.0 μL/g; ketamine:xylazine ratio = 7:1). The concentration of inhaled halothane was maintained at 1.5–2.0% during surgery. The depth of anesthesia was assessed by respiratory rate and responsiveness to tail pinch.

The same surgical procedure as described in our previous study (see Yazawa, 2014; Yazawa and Shioda, 2015) was then performed to prepare the decerebrate and arterially perfused *in situ* preparation. Mice were fixed in a supine position in a dissection chamber, and a median laparotomy was performed from the xiphoid to the lower abdomen. The stomach, small and large intestines, spleen, and pancreas as well as their dominant vessels were ligated and removed. Then, a thoracotomy was performed to allow us to directly visualize the heart and lungs, and both the pleura and the pericardium were removed after an intracardiac injection of 10 U/L heparin. The preparation was immediately submerged in Ringer’s solution infused with a 95% O_2_–5% CO_2_ gas mixture and maintained at 5–10°C to induce suspended animation. Ringer’s solution consisted of the following (in mM): 125 NaCl, 3 KCl, 24 NaHCO_3_, 1.25 KH_2_PO_4_, 1.25 MgSO_4_, 2.5 CaCl_2_, and 10 d-glucose, equilibrated with 95% O_2_–5% CO_2_ at pH 7.40–7.45 at room temperature (Chizh et al., 1997). After confirmation of cardiac arrest, a craniotomy was performed. Decerebration was performed with suction at the precollicular level. To prevent fluid from accumulating in the subcutaneous tissue, the skin was removed from the entire body. The bilateral lungs were cut at the level of the lobar bronchi and the apex of the left ventricle was incised.

After the mouse was transported to the recording chamber and then held in a supine position, a double-lumen catheter (Φ 1.0 mm, DL-AS-040; Braintree Scientific, MA, USA) was inserted into the heart through the incision in the left ventricle. To ensure that the perfusate entered the ascending aorta without backing up into the left ventricle, we modified the outer diameter of the tip of the catheter to be slightly larger than the inner diameter. Arterial perfusion was immediately started with carbogen-bubbled Ringer’s solution containing an oncotic agent (1.25–1.28% Ficoll-70), heparin (10–20 U/L), and penicillin–streptomycin–neomycin (50 U/L) at room temperature. Finally, the right atrium was incised to maintain the internal pressure of the heart at atmospheric pressure, and the incised part of the left ventricle was then sutured to secure the catheter in the ascending aorta.

After the preparation resumed spontaneous breathing, the muscle relaxant *d*-tubocurarine (2 μM) was added to the perfusate to induce immobilization. The left phrenic nerve (PHN) was identified at the diaphragm level, detached from blood vessels and connective tissues, and severed at the distal end. The left and right peripheral motor nerves were carefully detached at the knee level and severed at their distal ends. Although there was pronounced bradycardia at the initiation of perfusion, ventricular fibrillation never developed.

The same perfusion circuit system as described in our previous studies (see Yazawa, 2014; Yazawa and Shioda, 2015) was used to keep the preparations alive at room temperature. The perfusate, equilibrated with 95% O_2_–5% CO_2_ at the reservoir, was circulated via the perfusion circuit with a peristaltic pump (model 323 U pump, model 318MC pump head; Watson-Marlow, Wilmington, MA, USA), transfused into the aortic arch of the preparation through bubble traps and net filters (nylon net pore size, 20 μm), and then recycled from the recording chamber back to the reservoir. After the preparation resumed spontaneous breathing, the perfusion flow was always set to >5× the total blood volume (TBV) per minute at room temperature. TBV was calculated as 1/13 (g) of body weight according to the calculation methods described by Mitruka and Rawnsley (1981) and by Harkness and Wagner (1989). In addition, systemic blood pressure was monitored via the second lumen of a double-lumen catheter using a strain-gauge pressure transducer (Pressure Monitor BP-1, WPI, FL, USA). All chemicals used in this study were purchased from Sigma (St. Louis, MO, USA).

### Hindlimb preparation

Five female WT mice and 5 female *Irf8^−/−^* mice on a C57BL/6 background (Jackson Laboratories; aged 11–12 weeks), along with 10 male Swiss Webster mice (Taconic Laboratory; aged 6–51 days), were used to produce hindlimb preparations, which were obtained by transecting decerebrate and arterially perfused *in situ* preparations at the level of the fifth thoracic vertebra. In this preparation, a double-lumen catheter (NCV25GW-200 W; CMP Inc., Tokyo, Japan) was inserted into the descending aorta from the severed end of the thoracic aorta, and the thoracic aorta was ligated at the level of the 6th thoracic vertebra to prevent leakage of perfusate. Arterial perfusion was initiated at 5× TBV/min at room temperature. After spontaneous alternating and synchronous movements were observed in the left and right hindlimbs of the preparation, 1–2 μM *d*-tubocurarine was added to the perfusate, and the peripheral motor nerves were detached as described above.

### Extracellular recordings

Suction electrodes constructed of polyethylene tubing (PE 50; Becton, Dickinson and Company, Franklin Lakes, NJ, USA) were used to record neuronal discharge from the left PHN, left (L-PN), and right peroneal (R-PN), and left tibial nerves (L-TN) at room temperature. PHN discharge is an indicator of the output derived from the brainstem respiratory center (Barman and Gebber, 1976). PN and TN discharges are indicators of the outputs produced by the CPG network formed between the fourth lumbar and third sacral spinal segments and by the CPG network formed between the fourth lumbar and second sacral spinal segments, respectively. The change in systemic pressure is an indicator of changes in sympathetic tone derived from the cardiovascular center of the brainstem (Coleridge and Coleridge, 1980; Julius and Nesbitt, 1996). The resultant neurograms were amplified ×1,000, filtered at 1–3000 Hz, and digitized using a Digidata 1320A and a Clampex (Axon Instruments, Union City, CA, USA) at sampling rates of 10,000 Hz. All data were saved on the hard disk of a compatible computer for further analysis. Lab Chart 7 software (AD Instruments Inc., Colorado Springs, CO, USA) was used for analysis in this study.

### Data analysis

In this study, we used the same data analysis methods as described in our previous studies (see Yazawa and Shioda, 2015). L-PN, R-PN, and L-TN discharges were selected from a recorded sequence, and the integrated waveforms were used to evaluate the phase difference between the two motor nerves. Using methods of circular statistics described by Batschelet (1981), the phase difference between the peak amplitudes of the two neuronal discharges during discharge episodes in the L-PN and R-PN and the L-PN and L-TN were determined. In the phase-shift analysis, each cycle period of L-PN discharge during the discharge episode was measured. Subsequently, the time lag between L-PN and R-PN or L-TN discharges in the cycle period of the L-PN discharge was measured. The phase value was obtained by dividing the time lag between the L-PN and R-PN or L-TN discharges in the cycle period of the L-PN discharge. Each phase value was then multiplied by 360. The values were then plotted on a circle representing the phase difference of possible phases from zero to 360°. Phase values of zero and 360° are equivalent and reflect synchrony; in contrast, 180° represents alternation. The mean phase and the coupling ratio (*r*), which indicates the concentration of phase values around the mean, are shown by the direction and the length of the vector originating from the center of the circle. If the phases of two discharges are strongly coupled, then the phase values will be expected to be highly concentrated around the mean phase. The coupling was considered significant when the Rayleigh test, which determines whether the concentration *r* is sufficiently high to state that coupling was present (Batschelet, 1981), had a *p-*value <0.001. All data compressed to a sample rate of 20 Hz were used.

## Results

### Perfusion flow dependence of systemic pressure (black), L-PN (Red), R-PN (green), and PHN (gray) discharge

From the results regarding the dependence of systemic pressure and phrenic and peripheral motor nerve discharges under room temperature on the perfusion flow rate in a decerebrate and arterially perfused *in situ* preparation of Swiss Webster mice aged 14–31 days described in a previous work by one of the authors (Yazawa, 2014), the following phenomena were found to be induced: (i) Resumption of spontaneous breathing occurred within 15 min after the onset of perfusion at room temperature. (ii) If the perfusion flow rate was high enough to generate a systemic pressure of >30 mmHg, spontaneous respiration was initiated. Additionally, when the flow rate was set at >5× TBV/min, PHN discharge showed a pattern of increasing amplitude, and its frequency displayed a regular rhythm. (iii) As the flow rate increased further, each neuronal discharge transformed into a discharge episode of increasing frequency and duration, which occurred periodically. (iv) All discharge episodes derived from the three nerves were produced at the same time. (v) When the flow rate was set at >10× TBV/min, three neuronal discharge episodes clearly showed rhythmic discharge patterns. (vi) Small changes in systemic pressure were elicited during and after discharge episodes. In addition, (vii) although an increase in perfusion flow volume caused an increase in oxygen consumption as described in human extracorporeal circulation (Fox et al., 1982; Kirklin and Barratt-Boyes, 1993), increased metabolism also caused an increase in neural activity. The present study is the first to investigate whether the above phenomena, especially (iii) to (vii), are produced even in decerebrate and arterially perfused *in situ* preparations made from 11- to 12-week-old mice on a C57BL/6 background.

**Figure 1:**
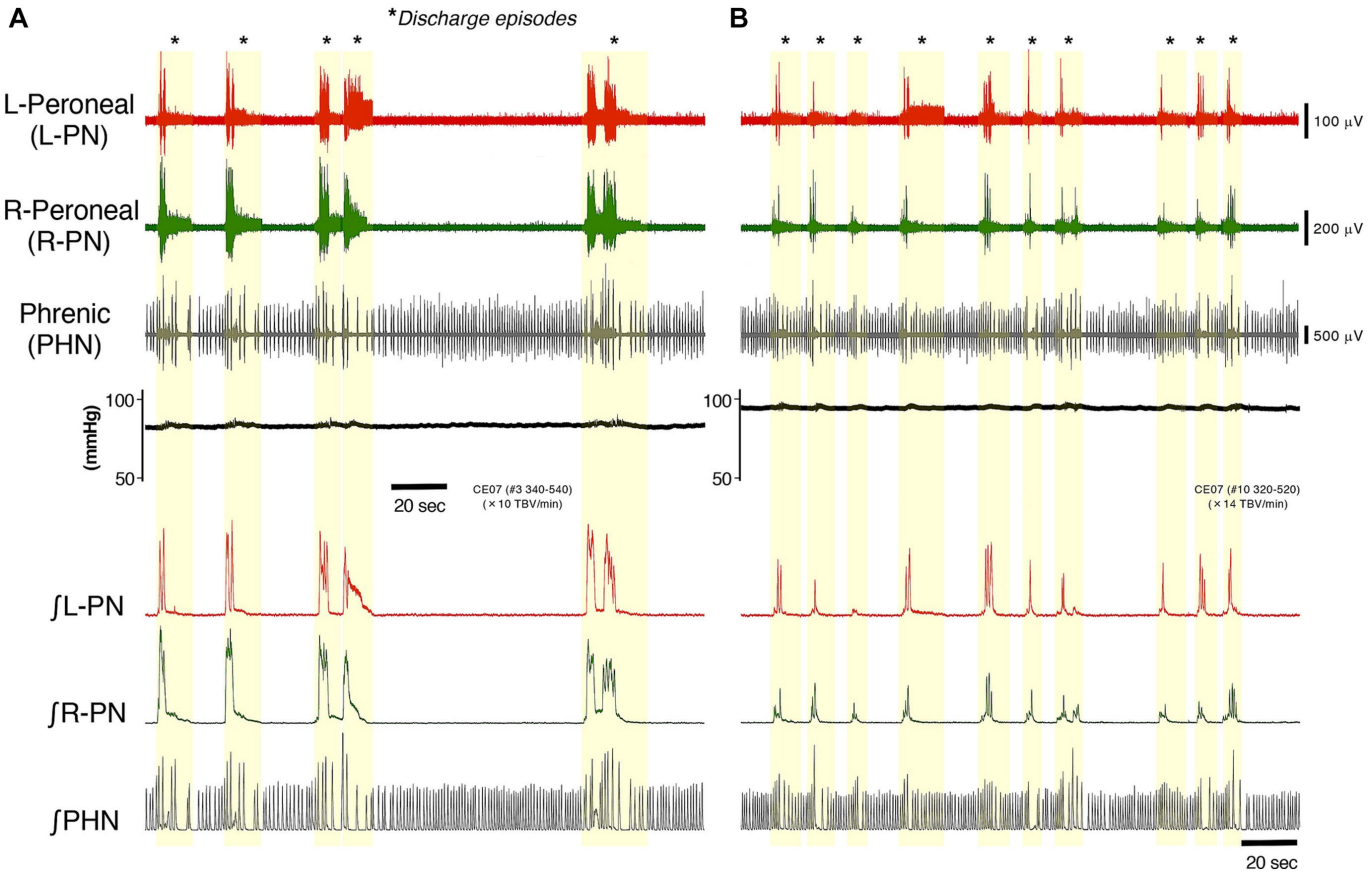
Figure represents typical examples of recordings showing the perfusion flow dependence of systemic pressure, L-PN (red) and R-PN (green), and PHN (gray) discharge at room temperature in decerebrate and arterially perfused *in situ* preparations made from mice on the C57BL/6 background aged 11–12 weeks. **(A,B)** Show the data collected on perfusion flow dependence at 10× and 14× TBV/min, respectively. Simultaneously, the systemic pressure (black) was monitored (upper). The integrated waveforms of the L-PN (∫ L-PN; red), R-PN (∫ R-PN; green), and PHN (∫ PHN; gray) discharges are shown in the lower panel. Asterisks show discharge episodes (yellow-shaded region). All data were obtained from the same preparation.

Figure 1 represents typical examples of recordings showing the perfusion flow dependence of systemic pressure, L-PN, R-PN, and PHN discharge at room temperature in decerebrate and arterially perfused *in situ* preparations made from adult C57BL/6 mice aged 11–12 weeks. At a high flow rate (>10× TBV/min), each nerve discharge transformed into a discharge episode of increasing frequency and duration, which occurred periodically. Figures 1A,B show the data collected on perfusion flow dependence at 10× and 14× TBV/min, respectively. Simultaneously, the systemic pressure was monitored (upper). The integrated waveforms of the L-PN (∫ L-PN), R-PN (∫ R-PN), and PHN (∫ PHN) discharges are shown in the lower panel. All data were obtained from the same preparation. Asterisks display discharge episodes (yellow-shaded region). The three nerve discharge episodes were produced at approximately the same time. At flow rates of >10× TBV/min, they showed rhythmic discharge patterns during discharge episodes. Several small systemic pressure changes were elicited during discharge episodes. In addition, the frequency of occurrence of the L-PN, R-PN, and PHN discharge episodes increased with increasing flow rates. Similar results to those shown in Figure 1 were reproduced in all the preparations made from mice from the C57BL/6 background aged 11–12 weeks (*n* = 5), indicating that a certain sympathetic tone resulting from an increase in flow rate activated the lumbar CPG network via descending pathways and initiated discharge episodes (see “A decerebrate and arterially perfused *in situ* preparation” section of Discussion).

### Discharge episodes in peripheral motor nerves and phase relationships between the L-PN (red) and R-PN (green) and L-PN (red) and L-TN (blue) rhythmic discharge episodes induced at high flow rates in decerebrate and arterially perfused *in situ* preparations made from adult C57BL/6 mice aged 11–12 weeks

In the decerebrate and arterially perfused *in situ* mouse preparations, a certain sympathetic tone resulting from an increase in flow rate is modulated when using high flow rates (>10× TBV/min) at room temperature because the preparation is exposed to a hyperoxic/normocapnic state. Modulated sympathetic tone activates the lumbar CPG network via descending pathways and generates discharge episodes and rhythmic neuronal discharge episodes, and locomotor-like activity is autonomously generated in the hindlimbs of the preparation (Yazawa, 2014). We next investigated the occurrence pattern of the discharge episodes in peripheral motor nerves and phase relationships between the L-PN/R-PN and L-PN/L-TN rhythmic discharge episodes, induced at high flow rates, in preparations made from adult C57BL/6 mice aged 11–12 weeks.

**Figure 2:**
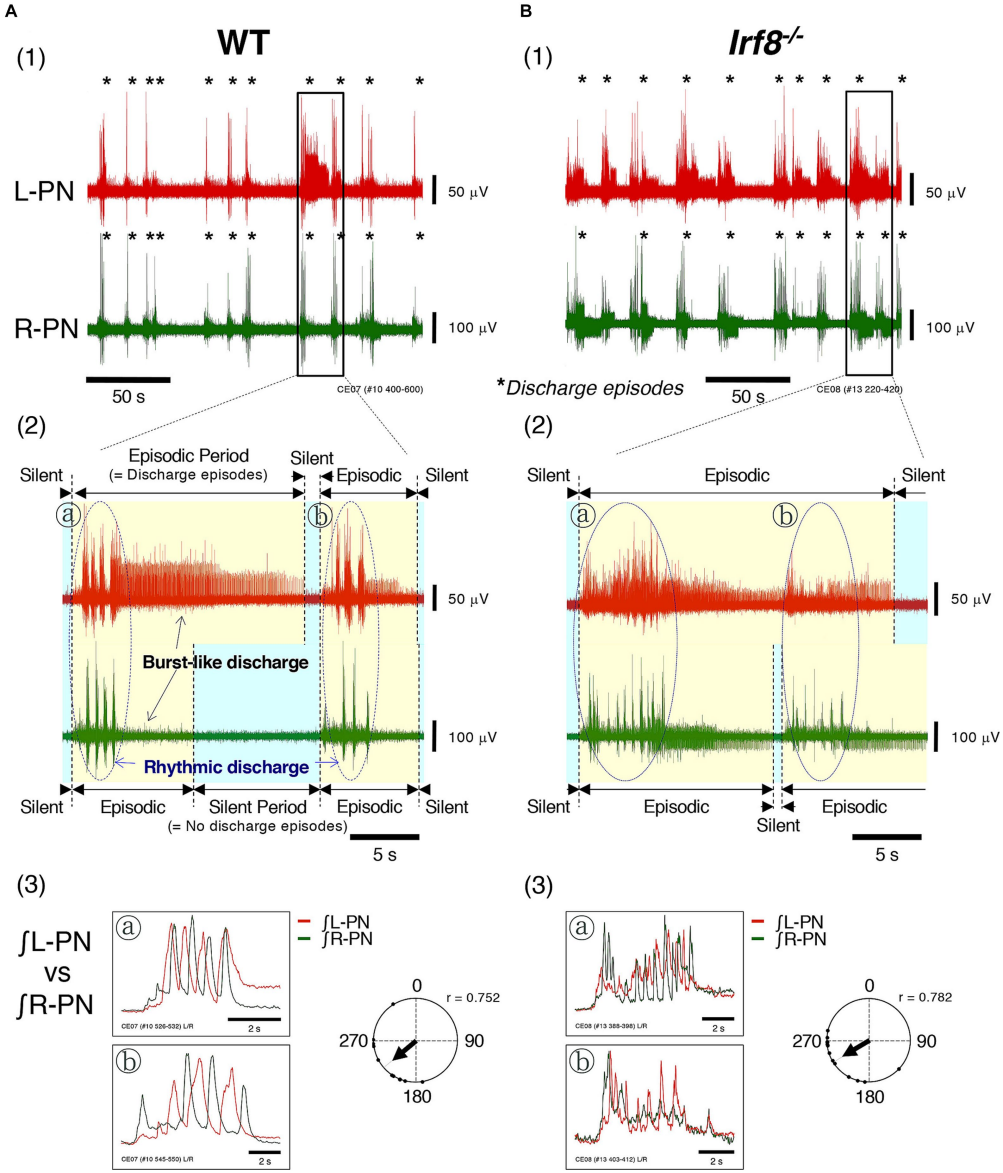
Figure shows the instances of discharge episodes and rhythmic neuronal discharge episodes from the bilateral peripheral motor nerves, induced at high flow rates (>10× TBV/min) at room temperature in decerebrate and arterially perfused *in situ* preparations made from adult C57BL/6 mice aged 11–12 weeks. **(A1,B1)** Show typical examples of neuronal discharges recorded from L-PN (red) and R-PN (green) at room temperature, induced at 14× and 16× TBV/min, in preparations made from adult WT and *Irf8^−/−^* C57BL/6 mice aged 11–12 weeks. Asterisks show discharge episodes. **(A2,B2)** present enlarged views of the L-PN (red) and R-PN (green) discharges surrounded by the rectangular regions of **(A1,B1)**, where neuronal discharge episodes on both sides became organized into ‘discharge episodes consisting of rhythmic and burst-like discharges (episodic periods; yellow-shaded regions)’ punctuated by periods of quiescence (silent periods; blue-shaded regions), and the bilateral neuronal discharge episodes were simultaneously repeated episodic and silent periods. **(A3,B3)** Display the integrated waveforms of the L-PN (∫ L-PN; red) and R-PN (∫ R-PN; green) discharges in regions ⓐ and ⓑ surrounded by dashed lines of **(A2,B2)**, where rhythmic rather than burst-like discharges occur. Each data point shown in **(A,B)** was obtained from the same preparation. Circular statics were used to determine the phase difference from 0 to 360° between the instances of the rhythmic discharges in the L-PN and R-PN discharge episodes (*n* = 5). The phase difference between the rhythmic discharges in the L-PN (red) and R-PN (green) of preparations made from WT and *Irf8^−/−^* mice was approximately 230° (*r* = 0.752) and approximately 240° (*r* = 0.782), respectively.

Figures 2A,B show the instances of discharge episodes and rhythmic neuronal discharge episodes from the L-PN and R-PN, induced at flow rates of 14× and 16× TBV/min at room temperature, in decerebrate and arterially perfused *in situ* preparations made from adult WT and *Irf8^−/−^* C57BL/6 mice. In the preparations from WT mice, the L-PN and R-PN discharge episodes became organized into ‘discharge episodes (episodic periods; yellow-shaded regions)’ consisting of rhythmic and burst-like discharges punctuated by periods of quiescence (silent periods; blue-shaded regions), with simultaneously repeated episodic and silent periods on both sides (Figures 2A1,2). In the preparations made from adult *Irf8^−/−^* mice, although the L-PN and R-PN discharge episodes also became organized into ‘discharge episodes (episodic periods; yellow-shaded regions)’ consisting of rhythmic and burst-like discharges punctuated by periods of quiescence (silent periods; blue-shaded regions), the bilateral neuronal discharge episodes were not necessarily simultaneously repeated episodic and silent periods (Figures 2B1,2. Figures 2A3,B3 display the integrated waveforms of the L-PN (∫ L-PN) and R-PN (∫ R-PN) discharges in regions ⓐ and ⓑ surrounded by dashed lines in Figures 2A2,B2, where rhythmic rather than burst-like discharges occurred. The phase difference between the peak of the integrated waveforms of the L-PN (∫ L-PN) and R-PN (∫ R-PN) rhythmic discharges in the preparations made from adult WT and *Irf8^−/−^* mice was approximately 230° (*r* = 0.752) and approximately 240° (*r* = 0.782), respectively. In both cases, the rhythm frequency of elicited left–right alternating discharges remained constant at 1–2 Hz. Similar results to those shown in Figure 2 were reproduced in all preparations made from adult WT (*n* = 5) and *Irf8^−/−^* mice (*n* = 5).

From the above, it was indicated that modulated sympathetic tone activated the lumbar CPG network via descending pathways and generated discharge episodes and rhythmic neuronal discharge episodes and that locomotor-like activity was autonomously generated in the hindlimbs of the preparations made from adult WT and *Irf8^−/−^* mice aged 11–12 weeks.

### Occurrence pattern of discharge episodes in peripheral motor nerves and phase relationships between L-PN (red) and R-PN (green) and between L-PN (red) and L-TN (blue) rhythmic discharge episodes induced by the application of rhythmogenic drugs at a certain flow rate in hindlimb preparations made from adult C57BL/6 mice aged 11–12 weeks

We applied rhythmogenic drugs such as serotonin (5-HT), N-methyl-D, L-aspartate (NMDA), dopamine (DA) and/or noradrenaline (NA) to hindlimb preparations at a certain flow rate and explored whether discharge episodes and neuronal discharge episodes resulting from lumbar CPG network activation, as shown in Figures 2A,B, were induced.

**Figure 3:**
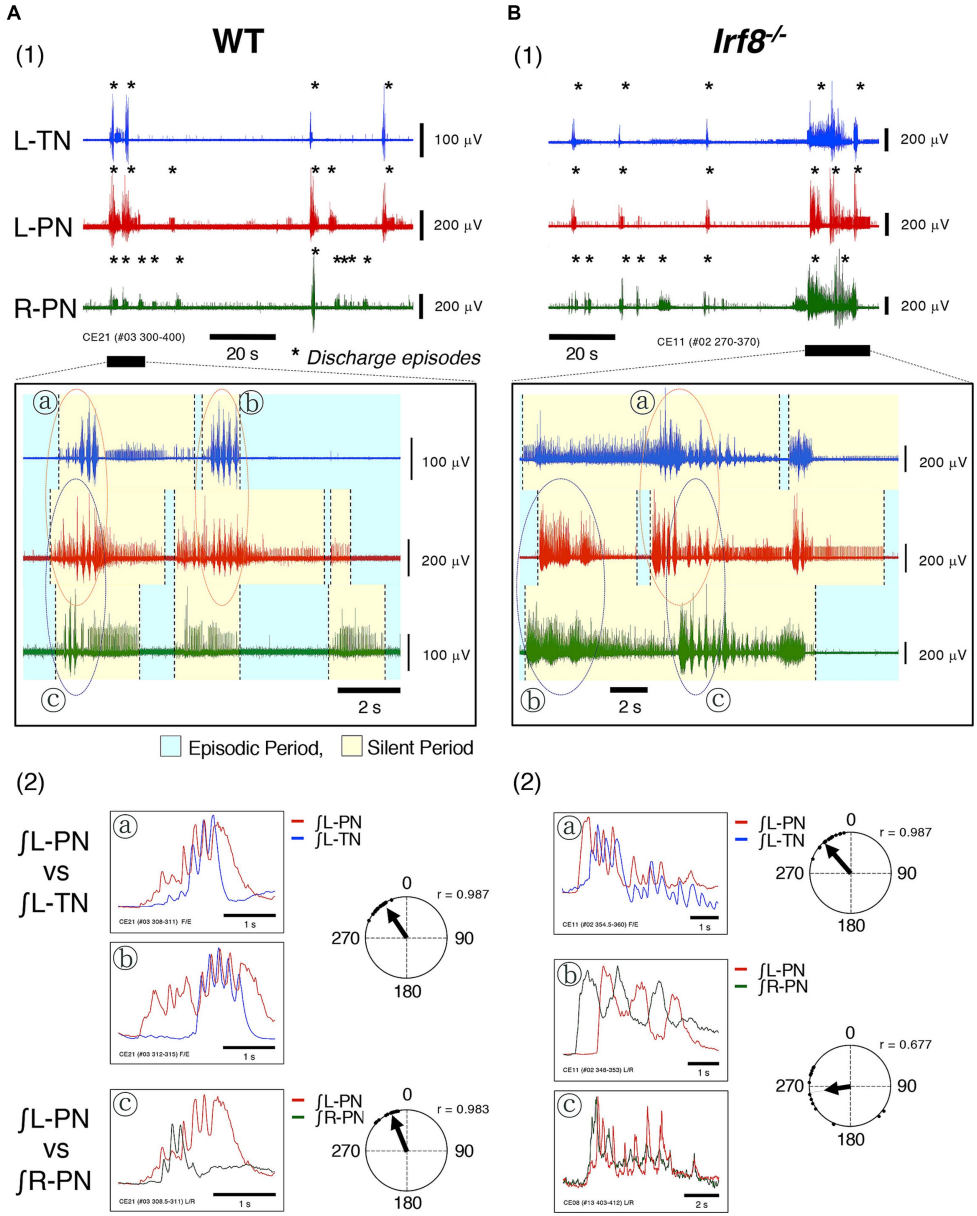
Figure shows typical examples of discharge episodes and rhythmic neuronal discharge episodes from the peripheral motor nerves induced by applying rhythmogenic drugs to hindlimb preparations made from adult C57BL/6 mice aged 11–12 weeks at room temperature. Each data point shown in **(A,B)** was obtained from the same preparation. **(A1,B1)** Show typical examples of neuronal discharges from L-TN (blue), L-PN (red), and R-PN (green), induced by applying 20 μM 5-HT + 10 μM NMDA +1 μM noradrenaline (NA), in hindlimb preparations made from adult WT and *Irf8^−/−^* C57BL/6 mice, in which the perfusion flow rate was set to 7.5× and 8× TBV/min, respectively. Asterisks show discharge episodes. The lower panels present expanded views of neuronal discharge episodes of the L-PN (red), R-PN (green) and L-TN (blue) in the underlined parts of **(A1,B1)**. Discharge episodes induced in these three nerves repeatedly displayed episodic periods with discharge episodes (yellow-shaded region) and silent periods without discharge episodes (blue-shaded region). Each occurrence pattern of discharge episodes in the L-PN (red) and R-PN (green) in **(A1,B1)** resembled that of discharge episodes shown in **(A2,B2)**. ⓐ and ⓑ, surrounded by dashed lines (red) in the lower panel of **(A1)**, show rhythmic discharges in the L-TN (blue) and L-PN (red). Ⓒ, surrounded by dashed lines (purple) in the lower panel of **(A1)**, shows rhythmic discharges in the L-PN (red) and R-PN (green). However, ⓐ, surrounded by dashed lines (red) in the lower panel of **(B1)**, shows rhythmic discharges in the L-TN (blue) and L-PN (red). ⓑ and Ⓒ, surrounded by dashed lines (purple) in the lower panel of **(B1)**, show rhythmic discharges in the L-PN (red) and R-PN (green). **(A2)** presents the integrated waveforms of the L-PN (∫ L-PN; red) and L-TN (∫ L-TN; blue) discharges in regions ⓐ and ⓑ in the lower panel of **(A1)** and the L-PN (∫ L-PN; red) and R-PN (∫ R-PN; green) discharges in region Ⓒ of the same lower panel. **(B2)** Displays the integrated waveforms of the L-PN (∫ L-PN; red) and L-TN (∫ L-TN; blue) discharges in region ⓐ in the lower panel of **(B1)** and the L-PN (∫ L-PN; red) and R-PN (∫ R-PN; green) discharges in regions ⓑ and Ⓒ of the same lower panel. Circular statics were used to determine the phase difference from 0 to 360° between the instances of rhythmic discharges in the L-PN (red) and L-TN (blue) discharge episodes and the L-PN (red) and R-PN (green) discharge episodes (*n* = 5). The phase difference between the rhythmic discharges in the L-PN (red) and R-PN (green) of preparations made from adult WT and Irf8^−/−^ C57BL/6 mice was approximately 335° (*r* = 0.983) and approximately 260° (*r* = 0.677), respectively. The phase difference between the rhythmic discharges in the L-PN (red) and L-TN (blue) of preparations made from adult WT and *Irf8^−/−^* C57BL/6 mice was approximately 325° (*r* = 0.987) and approximately 320° (*r* = 0.987), respectively.

Figures 3A1,B1 show typical examples of neuronal discharges from the L-TN, L-PN, and R-PN induced by the application of 20 μM 5-HT + 10 μM NMDA +1 μM NA to hindlimb preparations made from adult WT and *Irf8^−/−^* C57BL/6 mice. The perfusion flow rate was set at 7.5× and 8× TBV/min. Asterisks show discharge episodes. The lower panels present expanded views of neuronal discharge episodes of the L-PN, R-PN, and L-TN in the underlined parts of Figures 3A1,B1. It was found that discharge episodes induced in the three nerves repeatedly displayed episodic periods with discharge episodes (yellow-shaded region) and silent periods without discharge episodes (blue-shaded region) and that each occurrence pattern of discharge episodes in the L-PN and R-PN in Figures 3A1,B1 resembled that of discharge episodes shown in Figures 2A2,B2.

Figure 3A2 presents the integrated waveforms of the L-PN (∫ L-PN) and L-TN (∫ L-TN) discharges in regions ⓐ and ⓑ of the lower panel of Figure 3A1 and shows the L-PN (∫ L-PN) and R-PN (∫ R-PN) discharges in region Ⓒ of the same lower panel. Figure 3B2 displays the integrated waveforms of the L-PN (∫ L-PN) and L-TN (∫ L-TN) discharges in region ⓐ of the lower panel of Figure 3B1 and shows the L-PN (∫ L-PN) and R-PN (∫ R-PN) discharges of regions ⓑ and Ⓒ of the same lower panel. The phase difference between the rhythmic discharges in the L-PN and R-PN of the preparations made from adult WT and *Irf8^−/−^* C57BL/6 mice was approximately 335° (*r* = 0.983) and 260° (*r* = 0.677), respectively. In both cases, the rhythm frequency of elicited left–right alternating discharges remained constant at <4 Hz. Similar results to those shown in Figure 3 were reproduced in all preparations made from adult WT (*n* = 5) and *Irf8^−/−^* C57BL/6 mice (*n* = 5). On the other hand, the phase difference between the rhythmic discharges in the L-PN and L-TN of the preparations made from adult WT and *Irf8^−/−^* C57BL/6 mice was approximately 325° (*r* = 0.987) and 320° (*r* = 0.987), respectively. In both cases, the rhythm frequency of elicited flexion-extension-like discharges remained constant at <4 Hz. Similar results to those shown in Figure 3 were reproduced in all preparations made from adult WT (*n* = 5) and *Irf8^−/−^* C57BL/6 mice (*n* = 5).

Based on the results shown in Figures 2, 3, the pattern of occurrence of discharge episodes generated by lumbar CPG network activation differed in adult WT and *Irf8^−/−^* C57BL/6 mice.

### Developmental changes in the pattern of occurrence of discharge episodes caused by activation of the lumbar CPG network from the neonatal-juvenile stage (postnatal day < 14) to adulthood (postnatal day ≥ 14)

During the first 2 weeks of life, rodents acquire motor behaviors such as weight bearing and postural reflexes (Clarac et al., 1998). Mice can support their body weight by postnatal day 9 (P9), and many of the walking gait characteristics of mice at postnatal day 14 (P 14) are qualitatively similar to those of adult mice. In addition, the CPG network in the lumbar spinal cord is functionally mature by postnatal days 10–12 (P10–12) and is capable of generating locomotor-like activity (Jiang et al., 1999). During the postnatal period, microglia, which are the major phagocytes in the CNS promote apoptosis, eliminate apoptotic cells, prevent the overproduction of neurons by phagocytosing synapses and neurites, and contribute to the refinement of neuronal circuits (Salter and Stevens, 2017). As microglia mature, they alter their own transcriptional and functional identity as a result of changes in their density and morphology (Zusso et al., 2012). Brain microglia play a specialized role in microglial phagocytosis during development (Matcovitch-Natan et al., 2016; Hammond et al., 2019). However, IRF8-related microglia are normally absent in the lumbar cord dorsal horn of adult *Irf8^−/−^* mice (Masuda et al., 2012).

To understand the development of functional aspects of the lumbar CPG network caused by the absence of IRF8-related microglia, we examined developmental changes in the pattern of occurrence of discharge episodes caused by activation of the lumbar CPG network from the neonatal-juvenile period to adulthood using Swiss Webster mice (Taconic Laboratory) from 5 to 51 days of age.

**Figure 4:**
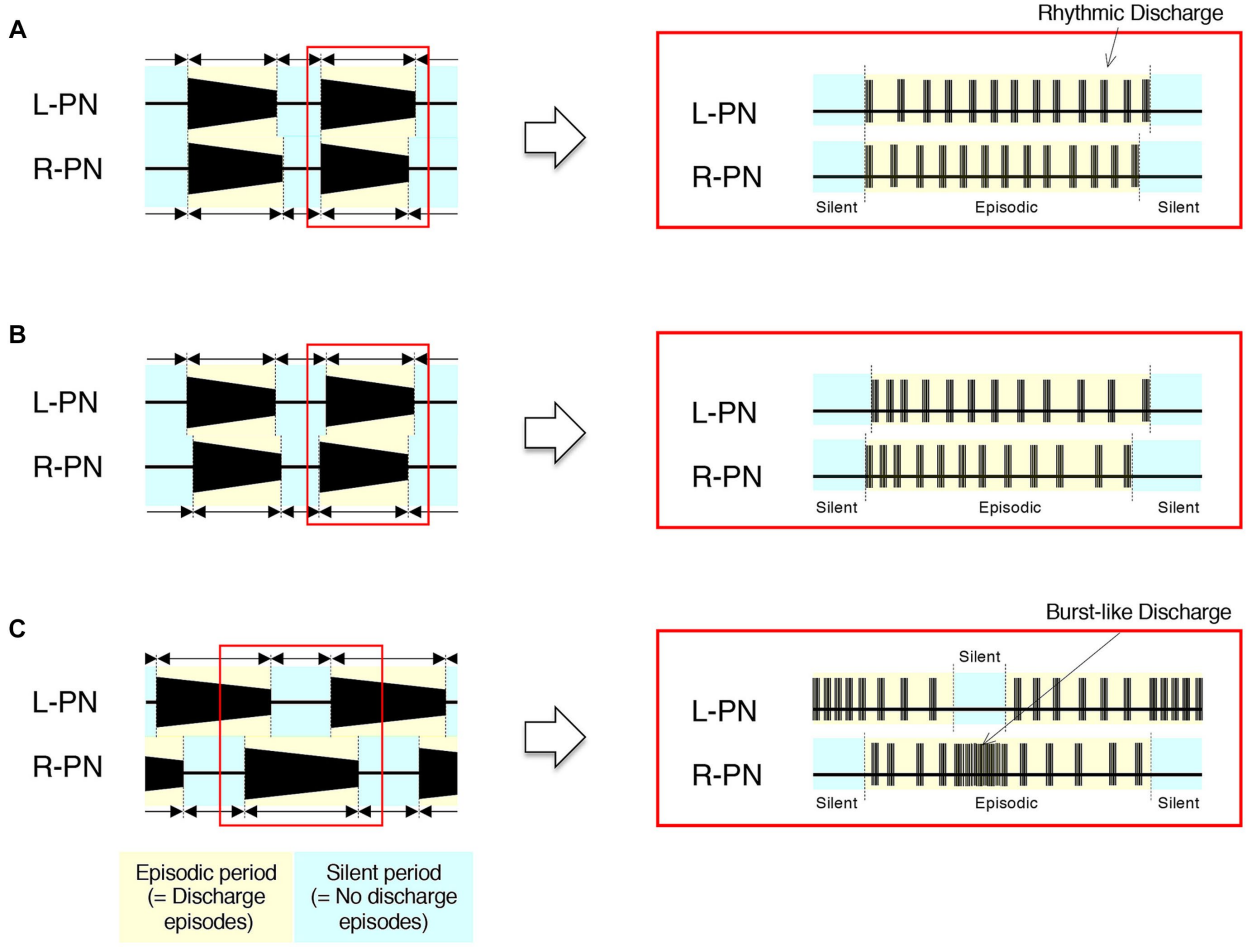
Schematics of developmental changes in the pattern of occurrence of discharge episodes (left) and neuronal discharge episodes (right panels) caused by activation of the lumbar CPG network from the neonatal-juvenile stage to adulthood at room temperature. **(A)** Schematics of the typical pattern of occurrence of discharge episodes (left) and the discharge patterns during the discharge episode (right panels) recorded from the L-PN and R-PN in decerebrate and arterially perfused *in situ* preparations made from mice after postnatal day five at high flow rates (>10× TBV/min). In this preparation, neuronal discharges became organized into ‘discharge episodes’ of increasing frequency and duration, punctuated by periods of quiescence as the flow rate increased, and the bilateral neuronal discharge episodes repeated episodic periods with discharge episodes and silent periods without discharge episodes. At a flow rate of <10× TBV/min, neuronal discharges during discharge episodes showed a burst-like discharge. However, at a flow rate of ≥10× TBV/min, they clearly showed rhythmic discharge episodes and represented a left–right alternating rhythmic discharge pattern beginning with synchronous discharge patterns. Regardless of age, the rhythm frequency of elicited left–right alternating discharges remained constant at 1–2 Hz. Similar results were reproduced in all 10 preparations made from Swiss Webster mice (Taconic Laboratory) aged 5–21 days (raw data not shown). **(B,C)** Represent schematics of the typical pattern of occurrence of discharge episodes (left) and the neuronal discharge patterns during the discharge episode (right panels) recorded from the L-PN and R-PN in hindlimb preparations made on postnatal days 14–51 and 6–8, respectively. In this preparation, neuronal discharge episodes consisting of a rhythmic and burst-like discharge (episodic periods) were generated by applying serotonin (5-HT), *N*-methyl-d, l-aspartate (NMDA), and dopamine (DA) or noradrenaline (NA) to the preparation at a flow rate of 5–7× TBV/min. In **(B)**, after administration of 20–140 μM 5-HT, 10–70 μM NMDA, and 1–5 μM NA, neuronal discharges became organized into episodes punctuated by periods of quiescence. Neuronal discharge episodes did not simultaneously occur on both sides. However, once the neuronal discharge episodes were initiated on both sides, they periodically and repeatedly generated episodic periods with discharge episodes and silent periods without discharge episodes. The frequency of left/right alternating discharge episodes in the L-PN and R-PN was <5 Hz. Similar results were reproduced in all hindlimb preparations (*n* = 5) (raw data not shown). In **(C)**, after administration of 40–120 μM 5-HT, 20–60 μM NMDA, and 40–450 μM DA or 1–3 μM NA, neuronal discharges became organized into episodes punctuated by periods of quiescence. In the preparations made from mice aged 6–8 days, the neuronal discharge episodes consisted of a rhythmic, burst-like, and then rhythmic discharge (episodic periods) and were always generated on either side. Neuronal discharge episodes on one side displayed a burst-like discharge whenever silent periods were produced on the other side. The frequency of left–right alternating discharge episodes in the L-PN and R-PN was <2 Hz. Similar results were reproduced in all hindlimb preparations (*n* = 5) (raw data not shown).

Figure 4 shows schematics of the pattern of occurrence of discharge episodes (left) and the discharge patterns during the episodes (right panels) recorded from the L-PN and R-PN in the preparations made from mice after postnatal day five at high flow rates (> 10× TBV/min) at room temperature. In the decerebrate and arterially perfused *in situ* preparations made from mice aged 5–21 days, the bilateral neuronal discharge cycled between episodic periods with discharge episodes and silent periods without discharge episodes. They clearly showed rhythmic discharge episodes and represented a left–right alternating rhythmic discharge pattern beginning with synchronous discharge patterns, and the frequency of elicited left–right alternating rhythmic discharges remained constant at 1–2 Hz (Figure 4A). Similar results to those shown in Figure 4A were reproduced in all 10 preparations (raw data not shown). In hindlimb preparations made from mice aged 14–51 days, after administration of 20–140 μM 5-HT, 10–70 μM NMDA, and 1–5 μM NA, each neuronal discharge transformed into a discharge episode of increasing frequency and duration, which occurred periodically, although bilateral neuronal discharge episodes did not occur at the same time. However, once the neuronal discharge episodes were initiated on both sides, they periodically and repeatedly generated episodic and silent periods. The frequency of left/right alternating discharge episodes in the L-PN and R-PN was <5 Hz (Figure 4B). Similar results were reproduced in all hindlimb preparations (*n* = 5) (raw data not shown). In hindlimb preparations made from mice aged 6–8 days, after administration of 40–120 μM 5-HT, 20–60 μM NMDA, and 40–450 μM DA or 1–3 μM NA, each neuronal discharge transformed into a discharge episode of increasing frequency and duration, which occurred periodically. The neuronal discharge episodes consisted of a rhythmic, burst-like, and then rhythmic discharge (episodic periods) and were always generated on either side. Neuronal discharge episodes on one side displayed a burst-like discharge whenever silent periods were produced on the other side. The frequency of left/right alternating discharge episodes in the L-PN and R-PN was <2 Hz (Figure 4C). Similar results were reproduced in all hindlimb preparations (*n* = 5) (raw data not shown).

Based on the results shown in Figures 2A2,B2, 3A1,B1, 4, the discharge episodes caused by lumbar CPG network activation in adult *Irf8^−/−^* C57BL/6 mice consisted of discharge episodes caused by activation of the newborn/juvenile and adult lumbar CPG networks, indicating that early-life immunodeficiency due to loss of IRF8 might interfere with the normal development of functions of the lumbar CPG network.

## Discussion

In this study, to understand the development of functional aspects of the lumbar CPG network in adult IRF8-deficient mice developing in the absence of IRF8-related microglia in the dorsal horn of the spinal cord, we used decerebrated and arterially perfused *in situ* preparations and extracellular recordings, investigated the developmental changes in the pattern of occurrence of discharge episodes generated by activation of the lumbar CPG network in Swiss Webster mice from the neonatal-juvenile stage to adulthood, and examined the pattern of occurrence of discharge episodes generated by activation of the lumbar CPG network in adult WT and *Irf8^−/−^* mice on the C57BL/6 background. The results indicated that the discharge episodes exerted by activation of the lumbar CPG network in adult *Irf8^−/−^* C57BL/6 mice consisted of the discharge episodes exerted by activation of the newborn-juvenile and adult lumbar CPG networks, suggesting the possibility that early-life immunodeficiency due to loss of IRF8 might interfere with the normal development of functions of the lumbar CPG network.

### Mechanism(s) of left and right rhythmic activity induced at high flow rates (≥10× TBV/min) at room temperature in the hindlimbs of decerebrated and arterially perfused *in situ* preparations

The decerebrate and arterially perfused *in situ* preparations survived via total artificial cardiopulmonary bypass for extracorporeal circulation, and the oxygen and ion components in the plasma needed for survival were supplied by blood vessels at room temperature.

In this preparation, afferent inputs from mechanosensors of the heart wall (Bishop et al., 1983; Hainsworth, 1991; Hines et al., 1994) to the cardiovascular center of the brainstem can be ignored because the right atrium was incised to maintain the internal pressure of the heart at atmospheric pressure. Afferent inputs from the stretch receptors of the lungs (Kalia and Sullivan, 1982; Hines et al., 1994) to the respiratory center of the brainstem can be ignored because of the removal of the lungs. In addition, afferent inputs from glomus type I cells on the carotid body that sense thermal changes (Alcayaga et al., 1993) to the respiratory center of the brainstem can be ignored because the preparation was maintained at room temperature. A peristaltic pump was used to provide pressure pulse waves to the baroreceptors of the preparation, as parasympathetic/sympathetic control of vascular resistance via the baroreflex is affected specifically by pulsatile rather than non-pulsatile flow (James and de Burgh Daly, 1970; Chapleau et al., 1989). Furthermore, the effect of the impulse from the central chemoreceptor, the pH/PCO_2_ sensor, on the respiratory center of the brainstem can be ignored because the pH of the perfusate was maintained within the physiological range before and after systemic perfusion (Loeschcke, 1982; O'Regan and Majcherczyk, 1982; Nattie, 1998; Ballantyne and Scheid, 2001). Therefore, the homeostasis of this preparation was maintained under the influence of afferent inputs from baroreceptors and peripheral chemoreceptors in the aortic arch and carotid sinus, along with central chemoreceptors distributed on the ventral medullary surface.

After the resumption of spontaneous breathing in the preparation, PHN discharge occurred in a pattern of increasing amplitude, while peripheral motor nerve discharge occurred in a pattern of decreasing amplitude hundreds of milliseconds after the occurrence of PHN discharges. When the flow rate was set at >10× TBV/min, each neuronal discharge transformed into a discharge episode of increasing frequency and duration, which occurred periodically. Discharge episodes in peripheral motor nerves on both sides displayed an alternating pattern of left–right discharge. The physiological condition of the preparation under this flow rate setting was considered to be as follows: Although the sympathetic tone of the preparation increased with increasing perfusion flow volume, the sympathetic tone of the preparation maintained in the hypothermic state was extremely low compared with that of animals maintained at normothermia. The preparation was susceptible to a hyperoxic state due to the high flow rate at room temperature. Thus, when a high flow rate was set, the sympathetic tone seen at the high flow rate (≥10× TBV/min) was easily modulated by afferent input from the peripheral chemoreceptors. Locomotor-like activity, produced by modulated sympathetic tone activating the lumbar CPG network via the spinal descending pathway, was observed in the hindlimbs of the preparation.

### Neural networks comprising the lumbar CPG network caused by IRF8-related microglial cell deficiency

Microglia, which are the major phagocytes in the CNS, contribute to the postnatal refinement of neuronal circuits by promoting apoptosis, eliminating apoptotic cells, and preventing the overproduction of neurons (Salter and Stevens, 2017). Brain microglia mature while altering their own transcriptional and functional identity as a result of changes in their density and morphology, and mature microglia play specialized phagocytic roles during development (Zusso et al., 2012; Matcovitch-Natan et al., 2016; Hammond et al., 2019). In addition, microglia settle in different brain regions at varying rates during development and express specific local gene profiles and phenotypes in adulthood. Thus, microglia display spatial heterogeneity in the brain (Schwarz et al., 2012; De Biase et al., 2017; Ayata et al., 2018). IRF8-related microglia in the lumbar cord dorsal horn were found to be absent in adult *Irf8^−/−^* mice, whereas they were low in adult wild-type mice (Masuda et al., 2012). We speculate, based on the results of the studies described above, that the absence of IRF8-related microglia in the dorsal horn of the spinal cord inhibited the postnatal refinement of the lumbar CPG network and interfered with the normal functional development of the lumbar CPG network. The candidates for the interneurons composing the CPG network that produces locomotor-like activity are a group of interneurons located in L1-L6 near the central canal and the medial middle zone (Kjaerulff et al., 1994). Some dorsally derived interneurons originating from the dorsal horn area migrate ventrally during development, and others migrate ventrally after development (Gross et al., 2002; Lu et al., 2015). Based on this finding and the results shown in Figures 2–4, we propose that immunodeficiency due to loss of IRF8 interferes with the normal development of the inhibitory and excitatory neural circuits and dorsally derived interneurons connecting the bilateral neural networks that constitute the lumbar CPG network.

## Data availability statement

The raw data supporting the conclusions of this article will be made available by the authors, without undue reservation.

## Ethics statement

The animal study was approved by the National Institute of Neurological Disorders and Stroke (NINDS) and the National Institute of Child Health and Human Development (NICHD)/National Institutes of Health (NIH) Animal Care and Use Committee. The study was conducted in accordance with the local legislation and institutional requirements.

## Author contributions

IY conceived and initiated the project, performed the experiments with mice, analyzed the data, steered the entire project, and wrote the manuscript. IY, YY, RY, and KO approved the final version to be published. All authors contributed to the article and approved the submitted version.
